# Bio-Inspired Spike-Timing-Dependent Plasticity Learning with Metal Halide Perovskites: Toward Artificial Synaptic Functionality

**DOI:** 10.1021/acsami.5c21545

**Published:** 2026-01-22

**Authors:** Mostafa Shooshtari, So-Yeon Kim, Saeideh Pahlavan, Teresa Serrano-Gotarredona, Juan Bisquert, Bernabé Linares-Barranco

**Affiliations:** † Instituto de Microelectrónica de Sevilla, IMSE-CNM, (CSIC Universidad de Sevilla), Av. Américo Vespucio 28, 41092 Sevilla, Spain; ‡ Instituto de Tecnología Química (ITQ), Universitat Politècnica de València-Consejo Superior de Investigaciones Científicas (UPV-CSIC), 46022 València, Spain

**Keywords:** halide perovskite memristor, spike-timing-dependent plasticity (STDP), neuromorphic computing, synaptic plasticity, noise robustness, triplet-STDP

## Abstract

Recent advances in neuromorphic engineering have sparked a convergence between nanotechnology and neuroscience, where emerging devices such as memristors are being explored to replicate fundamental learning mechanisms observed in the brain. One such mechanism, spike-timing-dependent plasticity (STDP), encodes synaptic changes based on the precise timing between pre- and postsynaptic spikes, and has been widely adopted in machine intelligence and computational neuroscience. In this work, we demonstrate that a halide perovskite memristor (Cs_3_Bi_2_I_6_Br_3_) can effectively simulate biologically plausible STDP dynamics. We fabricate and characterize the MHP-based device, and develop a dynamic physical model capturing its voltage- and history-dependent switching behavior. Using biologically inspired biphasic voltage pulses, the model replicates classic STDP characteristics including long-term potentiation (LTP), long-term depression (LTD), and the canonical asymmetric learning window. Further analysis shows that the memristor supports advanced features such as triplet-STDP and synaptic memory consolidation. Importantly, the STDP behavior remains stable across 100 independent trials with biologically realistic voltage noise, exhibiting less than 0.03% variation in synaptic weight. These results suggest that the inherent physical dynamics of halide perovskites enable bioinspired learning without external programming or algorithmic supervision. By bridging molecular-scale materials physics with spike-based computation, our findings lay the groundwork for implementing scalable, low-power, and noise-tolerant synaptic learning in next-generation neuromorphic computing systems.

## Graphical abstract

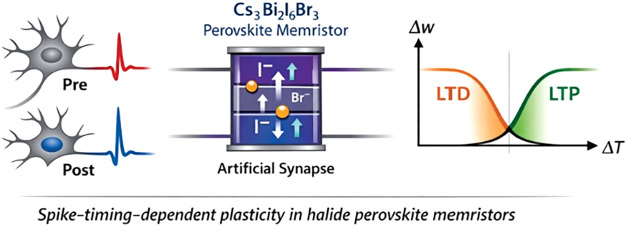

## 1 Introduction

The quest to understand and replicate the brain’s remarkable learning and memory capabilities has driven significant advancements in both neuroscience and neuromorphic engineering. One of the most fundamental mechanisms underlying synaptic plasticity, the ability of synapses to strengthen or weaken over time is Spike-Timing-Dependent Plasticity (STDP).^1,2^ STDP is a biologically inspired learning rule that governs how synaptic strength is modified based on the precise timing of pre and postsynaptic spikes. It refines Hebbian learning by introducing a temporal dependency: if a presynaptic spike precedes a postsynaptic spike within a critical time window, synaptic potentiation occurs; conversely, if the postsynaptic spike occurs first, synaptic depression is induced.^3,4^ This mechanism plays a crucial role in memory formation, learning, and adaptation in biological neural networks and serves as the foundation for neuromorphic computing models.

The development of memristors, two-terminal devices whose resistance depends on the history of applied voltage and current, has opened new avenues for implementing synaptic plasticity in hardware.^5–8^ First theorized by Leon Chua in 1971 and later realized in nanoscale devices, memristors exhibit properties that closely mimic biological synapses, making them ideal candidates for neuromorphic computing.^9–11^ Their ability to modulate synaptic weights through resistance changes offers a compact, energy-efficient, and analog approach to synaptic adaptation, distinguishing them from conventional CMOS-based implementations. Researchers have successfully demonstrated STDP-like learning in artificial synapses using memristors, bridging the gap between neuroscience and nanotechnology.^1,12–15^

While traditional memristive materials, such as titanium dioxide (TiO_2_) and amorphous silicon, have shown promise, they often face limitations in terms of energy efficiency, scalability, and tunability. This has led to the exploration of alternative materials, among which perovskite-based memristors have emerged as a particularly exciting candidate. Perovskites, a class of materials with the general formula ABX_3_, exhibit exceptional electronic and ionic properties, including high ion mobility, tunable resistance states, and sensitivity to external stimuli such as light and temperature. These characteristics make perovskite memristors uniquely suited for emulating the dynamic and adaptive behavior of biological synapses.^16–20^

Halide perovskite memristors, in particular, offer several advantages over traditional metal-oxide-based memristors. Their low processing temperatures, high tunability of electrical properties, and strong ion migration effects closely resemble biological synaptic behavior, enhancing their potential for neuromorphic applications.^21^ Perovskite memristors exhibit multilevel resistive switching, enabling finer control over synaptic weight updates crucial for implementing complex learning rules. Their high ion mobility facilitates rapid resistance state transitions, allowing for faster and more biologically realistic STDP responses. Additionally, the ability to engineer perovskite compositions provides flexibility in optimizing device performance for specific neuromorphic applications.^18,22^

The use of MHP­(Cs_3_Bi_2_I_6_Br_3_) perovskite memristors in neuromorphic systems offers multiple advantages. First, Cs_3_Bi_2_I_6_Br_3_ is a lead-free metal halide perovskite, addressing environmental and toxicity concerns associated with traditional lead-based perovskites. Second, it exhibits stable and tunable resistive switching behavior, enabling precise control over multilevel synaptic weights, which are crucial for complex learning tasks. Third, its high ion mobility and defect-tolerant nature facilitate rapid and energy-efficient synaptic updates, enhancing the speed and adaptability of neuromorphic computing systems. The high ion mobility in Cs_3_Bi_2_I_6_Br_3_ primarily arises from the migration of halide anions (I^–^ and Br^–^), which can readily drift under an external electric field and modulate local defect states. This anion migration leads to reversible formation and rupture of halide vacancy filaments, governing the resistive switching and STDP dynamics. In contrast, Cs^+^ and Bi^3+^ ions exhibit much lower mobility due to stronger lattice binding, making their contribution to switching negligible under the low-voltage conditions applied here.^23,24^ Additionally, Cs_3_Bi_2_I_6_Br_3_ is solution-processable, allowing for cost-effective, large-scale fabrication while maintaining excellent stability under ambient conditions, making it a promising candidate for next-generation brain-inspired architectures.^25^

In this work, we explore the potential of perovskite memristors to implement STDP, a cornerstone of synaptic plasticity. By developing a custom STDP model tailored to the unique properties of perovskite materials, we aim to provide new insights into the mechanisms underlying synaptic learning and to demonstrate how perovskite memristors can be used to build next-generation neuromorphic systems. Our investigation includes a comparative analysis of their advantages over conventional memristors, an examination of their spike-dependent plasticity behavior, and circuit-level simulations to validate their effectiveness in neuromorphic computing applications. By integrating perovskite memristors into STDP-based learning models, we seek to advance the development of more efficient and biologically inspired artificial neural networks, bridging the gap between biological and artificial intelligence.

## 2 Experimental Section

### 2.1 Device Fabrication

The memristor devices were fabricated using a layered structure composed of Indium Tin Oxide (ITO) glass, a metal halide perovskite (MHP) layer (Cs_3_Bi_2_I_6_Br_3_), and a silver (Ag) top electrode. The ITO glass substrates were cleaned via sonication in deionized water (DIW) with detergent, acetone, and isopropyl alcohol (IPA) for 15 min each, followed by drying with an air-gun.

The MHP precursor solution with 0.3 M concentration was prepared by dissolving cesium bromide (CsBr) and bismuth­(III) iodide (BiI_3_) in a 3:2 molar ratio in a mixed solvent of N,N-dimethylformamide (DMF) and dimethyl sulfoxide (DMSO). The solution was filtered using a PTFE-H syringe filter (hydrophilic, 0.22 μm pore size). Prior to deposition, the ITO substrates were treated with UV-Ozone (UVO) for 30 min to enhance surface properties. Thirty μL of the MHP precursor solution was spin-coated onto the ITO substrates at 1000 rpm for 10 s, followed by 5000 rpm for 30 s. After 10 s at the second spin-coating step, diethyl ether was dripped onto the wet film to induce crystallization. The film was then annealed at 100 °C for 15 min, resulting in an orange-red MHP layer. All fabrication steps were performed in an argon-filled glovebox to prevent oxidation. Finally, a 100 nm thick Ag top electrode was deposited via thermal evaporation using a dot-shaped shadow mask. Figure S1 is the schematic flowchart illustrating the fabrication process of the MHP-memristor device.

### 2.2 Device Characterization

The structural and morphological characteristics of the Cs_3_Bi_2_I_6_Br_3_ films were thoroughly analyzed to evaluate the quality of the active layer. The crystalline structure of the resulting film was characterized by X-ray diffraction (XRD) using a CUBIX XRD DY0822 diffractometer equipped with a Cu Kα radiation source (λ = 1.54056 Å). The XRD pattern, recorded over a 2θ range of 10–70° with a step size of 0.02°, confirmed a trigonal *P*3̅*m*1 crystal structure, consistent with the calculated powder XRD in the previous reports (Figure 1a). The XRD pattern of Cs_3_Bi_2_I_6_Br_3_ film exhibits the characteristic reflections of the trigonal structure, including the multiplet of (101), (110), (111), (112), (202), and (122) peaks in the 14–32° region, in agreement with previously reported powder and single-crystal data for this composition. These peak positions match the expected lattice contraction induced by Br incorporation, as documented in earlier structural studies.^26–28^ The film thickness and surface morphology were further examined by field-emission scanning electron microscopy (FESEM) and atomic force microscopy (AFM), respectively (Figure 1b,c). The cross-sectional FESEM image reveals an active layer thickness of approximately 400 nm, while the AFM analysis (tapping mode, scan area: 2.0 μm^2^) shows a surface roughness (Rq) of 9.59 nm, indicating a compact, uniform, and smooth film well adhered to the ITO bottom electrode. These results confirm that the fabricated MHP layer possesses high structural integrity, suitable for reliable memristive performance.

**Figure 1:**
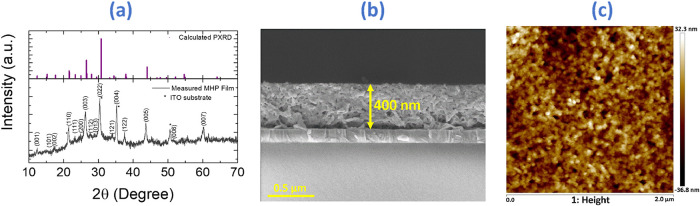
(a) XRD pattern of the Cs_3_Bi_2_I_6_Br_3_ film deposited on an ITO substrate with calculated powder XRD. (b) Cross-sectional SEM image of the Cs_3_Bi_2_I_6_Br_3_ memristive film showing a uniform active layer with an approximate thickness of about 400 nm on the ITO substrate. (c) AFM topography image of the same film obtained in tapping mode over a 2.0 μm^2^ area.

The fabricated memristor devices were characterized electrically using a probe station in dark conditions. Each memristor consisted of a 2.0 cm × 1.5 cm ITO/glass substrate with 200 μm diameter Ag electrodes. Electrical measurements were conducted using a KEYSIGHT B1500A semiconductor analyzer, which applied voltage to the top Ag electrode while the ITO bottom electrode was grounded.

Figure 2a shows the *I–V* characteristics were measured using a direct current (dc) voltage sweep sequence of 0 V → +1.0 V → 0 V → −1.5 V → 0 V at a compliance current of 1 mA with scan rate of 100 V/s. The device exhibited bipolar resistive switching with an initial forming step followed by stable switching cycles during 30 consecutive cycling tests. The SET process (HRS to LRS) occurred in the positive voltage region, while the RESET process (LRS to HRS) occurred in the negative region. To exclude the possibility that the observed polarity originates from Ag-related electrochemical metallization, we confirmed that devices fabricated with an inert Au top electrode exhibit the same bipolar switching behavior (see Supporting Figure S2). To assess cell-to-cell and device-to-device reproducibility, *I–V* sweeps were recorded from 50 different cells in one mother device as shown in Figure 2b and 18 different devices fabricated under the same conditions as shown in Figure 2c. Box plots of the high resistance state (HRS), low resistance state (LRS), and ON/OFF ratio read at +0.1 V are showed in Figure S3. Figure S3a,b is extracted from cell-to-cell reproducibility test and S2c is from device-to-device reproducibility test. An average HRS of 4.93 × 10^5^ Ω, LRS of 1.95 × 10^2^ Ω, and an ON/OFF ratio of 2.67 × 10^3^ at the forming step in Figure S3a. At the switching step, the respective values were 3.21 × 10^3^ Ω, 1.57 × 10^2^ Ω, and 1.97 × 10^1^ Ω in Figure S3b. Further device-to-device reproducibility tests were conducted on 18 different devices, confirming stable resistive switching in Figures 2c and S2c. An average HRS of 4.64 × 10^3^ Ω, LRS of 2.10 × 10^2^ Ω, and an ON/OFF ratio of 2.37 × 10^1^ at the forming step in Figure S3c. The low variability is statistically supported by the cumulative probability distributions (Figure S3d–f), which exhibit sharp transitions and narrow spreads, corroborating the high uniformity of the switching characteristics. The device durability was investigated in Figure S4 for 10^3^ consecutive cycling tests, in which the HRS and LRS were read at +0.1 V with the applied voltage of +1.0 V for SET process and −1.5 V for RESET process. Device stability was examined over 7 days under ambient conditions with over 60% humidity and room temperature. As it clear from Figure 2d while a slight increase in current was observed due to degradation, the bipolar resistive switching behavior remained intact. Continuous *I–V* sweeps at different scan rates (1000 V/s to 1 V/s) were performed to analyze dynamic switching characteristics as shown in Figure 2e.

**Figure 2:**
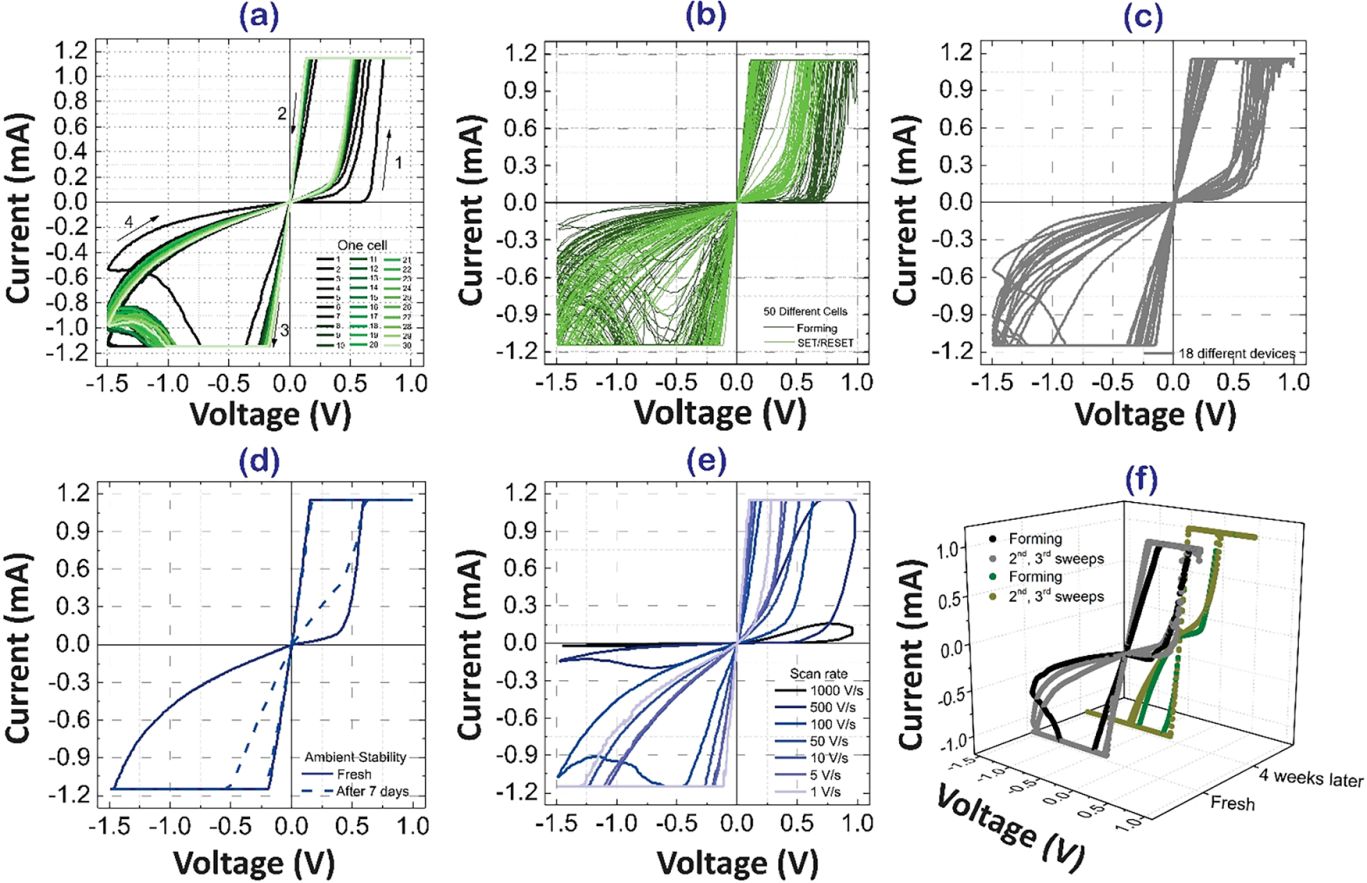
Structural and electrical characterization of Cs3Bi2I6Br3-based memristors at compliance current of 1 mA. (a) *I–V* characteristics of the memristor devices showing bipolar resistive switching behavior with a compliance current of 1 mA. The SET process occurs in the positive voltage region, while the RESET process is observed in the negative region. (b) Cell-to-cell reproducibility. *I–V* curves for forming and SET/RESET processes collected from 50 different cells in one mother device. (c) Device-to-device reproducibility. *I–V* curves for random cycles collected from 18 devices in different batches. (d) Stability of the memristor devices over 7 days under ambient conditions with over 60% humidity and room temperature, showing preserved bipolar resistive switching behavior despite a slight increase in current due to degradation. (e) Dynamic switching characteristics at different scan rates (1000 V/s to 1 V/s). (f) Long-term environmental stability. The *I–V* characteristics measured after 4 weeks of ambient exposure confirm the retention of bipolar resistive switching with negligible performance degradation.

The *I–V* characteristics measured after 4 weeks of ambient exposure without any encapsulation of the device show that the device retains its bipolar resistive switching behavior, as evident from the distinct hysteresis loops in Figure 2f. The negligible degradation in electrical performance over time clearly indicates environmental stability of the device. This long-term retention of switching characteristics under ambient conditions highlights the potential reliability and robustness of the fabricated memory device for practical nonvolatile memory and neuromorphic applications.

## 3 Modeling

### 3.1 MHP Memristor Model

Memristors, as theorized by Chua, are devices whose resistance depends on the history of applied voltage and current, making them ideal for mimicking synaptic behavior in neuromorphic systems.^29^ A general model for voltage-controlled memristors can be described by the following equations:^30^

1 $$I_{\mathrm{tot}}=C_{m}\frac{dV}{dt}+G\left(V,x_{i}\right)$$

2 $$\tau_{k}\frac{dx_{i}}{dt}=H\left(V,x_{i}\right)$$

Here, *I*_tot_ is the total current, *C*_m_ is the intrinsic capacitance, *V* is the applied voltage, and *x*_*i*_ represents the internal state variables of the memristor. The functions *G*(*V*,*x*_*i*_) and *H*(*V*,*x*_*i*_) describe the conduction and adaptation dynamics, respectively, while τ_*k*_ is the kinetic time constant governing the state variable’s evolution.

For MHP (metal halide perovskite) memristors, the conductance *G* varies between a minimum value *g*_*L*_ and a maximum value *g*_*H*_, controlled by a state variable *x* that ranges from 0 to 1.^31,32^ The total current is expressed as

3 $$I_{\mathrm{tot}}=C_{m}\frac{du}{\mathrm{dt}}+\left[g_{L}+\left(g_{H}-g_{L}\right)x\right]u$$

Here, *u* represents the internal voltage across the memristor, accounting for any small series resistance. The state variable *x* determines the conductance of the device, with *x* = 1 corresponding to the highest conductance *g*_*H*_ and *x* = 0 to the lowest conductance *g*_*L*_. The steady-state value of *x* as a function of voltage is given by a sigmoidal function:^31,32^

4 $$x_{\mathrm{ss}}\left(u\right)=\frac{1}{1+e^{-\left(u-V_{\mathrm{th}}\right)/V_{m}}}$$

where *V*_th_ is the threshold voltage and *V*_m_ controls the steepness of the transition.^31,32^ The time evolution of *x* is governed by

5 $$\tau_{x}\left(u\right)\frac{dx}{dt}=x_{\mathrm{SS}}\left(u\right)-x$$

Here, τ_*x*_(*u*) is a voltage-dependent relaxation time that determines how quickly *x* approaches its steady-state value. For MHP memristors, τ_*x*_ (*u*) is modeled as^31,33^

6 $$\tau_{x}\left(u\right)=\frac{\tau_{\max}+\tau_{\min\mathrm{OFF}}e^{-\left(u-V_{\mathrm{OFF}}\right)/V_{-}}}{1+e^{-\left(u-V_{\mathrm{OFF}}\right)/V_{-}}}-\frac{\tau_{\max}-\tau_{\min\mathrm{ON}}}{1+e^{-\left(u-V_{\mathrm{ON}}\right)/V_{+}}}$$

This equation accounts for the maximum (τ_max_) and minimum (τ_min OFF_ and τ_min ON_) relaxation times, as well as the threshold voltages *V*_OFF_ and *V*_ON_ for the RESET and SET processes, respectively. The ideality factors *V*_–_ and *V*_+_ further refine the voltage dependence of the relaxation time.

### 3.2 Verilog-A Implementation

To simulate the MHP memristor in circuit design software like Cadence, the model eqs 3–6 were implemented in Verilog-A, a hardware description language for analog and mixed-signal systems. Verilog-A allows for the creation of custom models that capture the behavior of complex devices, such as memristors, within a SPICE-based simulation environment.^34–36^

The Verilog-A code defines two virtual nodes to handle the derivative terms in the model.^34^ The current through the memristor depends on its conductance, the applied voltage, and the internal state variable *x*_SS_, which is modeled as a sigmoidal function. The relaxation time τ_*x*_(*u*) governs the dynamic transition of *x* between states, enabling the memristor to exhibit both volatile and nonvolatile behavior depending on the applied voltage. Equations 3–6 describe the behavior of the memristor using several parameters. Table 1 provides the definitions, values, and units of these parameters. By analyzing the shape and characteristics of the IV curve at different scan rates and maximum applied voltages, as shown in the experimental measurements in Figure 2, key parameters can be extracted.

**Table 1:** Definition, Unit, and Value of Parameters Describing MHP Memristor

parameter	definition	unit	value
*C*_m_	internal device capacitance	(nF)	0.20
*g*_*L*_	low conductance	(mS)	0.56
*g*_*H*_	high conductance	(mS)	12.10
*V*_th_	threshold voltage	(V)	0.00
*V*_m_	voltage modulation factor	(V)	0.05
τ_max_	maximum state transition time	(s)	500
*V*_OFF_	voltage offset for state transition	(V)	–0.30
*V*_–_	voltage offset factor for state transition	(V)	0.05
τ_min OFF_	minimum offset state transition time	(s)	0.08
*V*_ON_	voltage onset for state transition	(V)	0.03
*V*_+_	voltage onset factor for state transition	(V)	0.02
τ_min ON_	minimum onset state transition time	(s)	0.01

By implementing this model in Cadence, we were able to replicate the experimental results of MHP-based memristors, including their bipolar resistive switching and voltage-dependent relaxation dynamics. This approach provides a powerful tool for analyzing the memristor’s behavior in various circuit configurations and operational scenarios, paving the way for its application in neuromorphic computing and adaptive systems. As shown in Figure S5, the direct comparison of simulated and experimental *I–V* curves confirm the accuracy of the parameters in Table 1 in reproducing device dynamics, further supporting the model’s validity.

## 4 Results and Discussion

Figure S6 previews a synaptic junction, illustrating the connection between presynaptic and postsynaptic neurons. Neurons communicate with each other at junctions called synapses, where one neuron sends a message to a target neuron. Most synapses are chemical, relying on neurotransmitters for communication, while others are electrical, allowing ions to flow directly between cells. In a chemical synapse, an action potential triggers the presynaptic neuron to release neurotransmitters, which then bind to receptors on the postsynaptic cell, influencing its likelihood of firing an action potential. Synapses are typically formed between the axon terminals of the sending neuron and the cell body or dendrites of the receiving neuron. A single axon can have multiple branches, enabling it to form synapses with various postsynaptic cells, and a single neuron can receive thousands of synaptic inputs from different presynaptic neurons. Inside the axon terminal, there are synaptic vesicles filled with neurotransmitter molecules. Between the axon terminal of the presynaptic neuron and the postsynaptic cell membrane is a small gap called the synaptic cleft. When a neurotransmitter binds to its receptor on the receiving cell, it causes ion channels to open or close, leading to a localized change in the membrane potential, the voltage across the membrane of the receiving cell.^37,38^

The presynaptic neuron transmits an action potential, *V*_mem–pre_ (*t*), through its axon to the synaptic junction. Neural spikes are represented by membrane voltages across the cellular membrane, where *V*_mem–pre_ (*t*) = *V*_pre+_ – *V*_pre–_ (the potential difference between the outside and inside of the presynaptic cell), and similarly for the postsynaptic neuron, *V*_mem–pos_ (*t*) = *V*_pos+_ – *V*_pos–_. During a spike, these membrane voltages can reach hundreds of millivolts, opening and closing selective molecular channels that regulate the flow of ions and other substances across the membrane. In parallel, synaptic vesicles inside the presynaptic neuron, which contain neurotransmitters, fuse with the membrane and release their contents into the synaptic cleft, the small intercellular space between the pre- and postsynaptic neurons.

The released neurotransmitters bind to receptors on the postsynaptic membrane, causing changes in its membrane conductivity. This contributes to the cumulative effect of spikes from multiple presynaptic neurons, which ultimately triggers a new spike in the postsynaptic neuron. Each synapse is characterized by a synaptic weight *w* which determines the influence of a presynaptic spike on the postsynaptic neuron. This synaptic weight can be viewed as a structural parameter of the synapse, such as the number or size of neurotransmitter packets released during a presynaptic spike, or the amount of substances that influence the synaptic efficacy. Synaptic weight is considered nonvolatile and analog in nature, but it evolves over time based on the spiking activity of both the pre- and postsynaptic neurons.^39^

STDP extends this concept by describing the synaptic weight change as a function of the precise timing between pre- and postsynaptic spikes. The synaptic weight change Δ*w*, is a function of the time difference Δ*T* = *t*_pos_ – *t*_pre_, where *t*_pre_ and *t*_pos_ are the times at which the pre- and postsynaptic spikes occur, respectively. In STDP, if a presynaptic spike occurs shortly before a postsynaptic spike (a causal relationship), the synaptic weight increases, potentiating the synapse. Conversely, if the postsynaptic spike occurs before the presynaptic spike (an anticausal relationship), the synaptic weight decreases, leading to synaptic depression. This time-dependent learning mechanism allows for the precise tuning of synaptic strengths based on the relative timing of neuronal activity.

The shape of the STDP function spk­(t) can be interpolated from experimental data obtained by Bi and Poo,^40^ as illustrated in Figure 3a. For a positive Δ*T* (indicating that the presynaptic spike plays a significant role in generating the postsynaptic spike), there will be an increase in synaptic weight (Δ*w* > 0), and this effect becomes stronger as the magnitude of Δ*T* (|Δ*T*|) decreases. For a negative Δ*T* (indicating that the presynaptic spike is less relevant for the postsynaptic spike), the synaptic weight will decrease (Δ*w* < 0), with the depression being stronger as |Δ*T*| decreases. Bi and Poo found that there is an asymmetric critical window for Δ*T*, about 40 ms, within which synaptic modification can occur.

**Figure 3:**
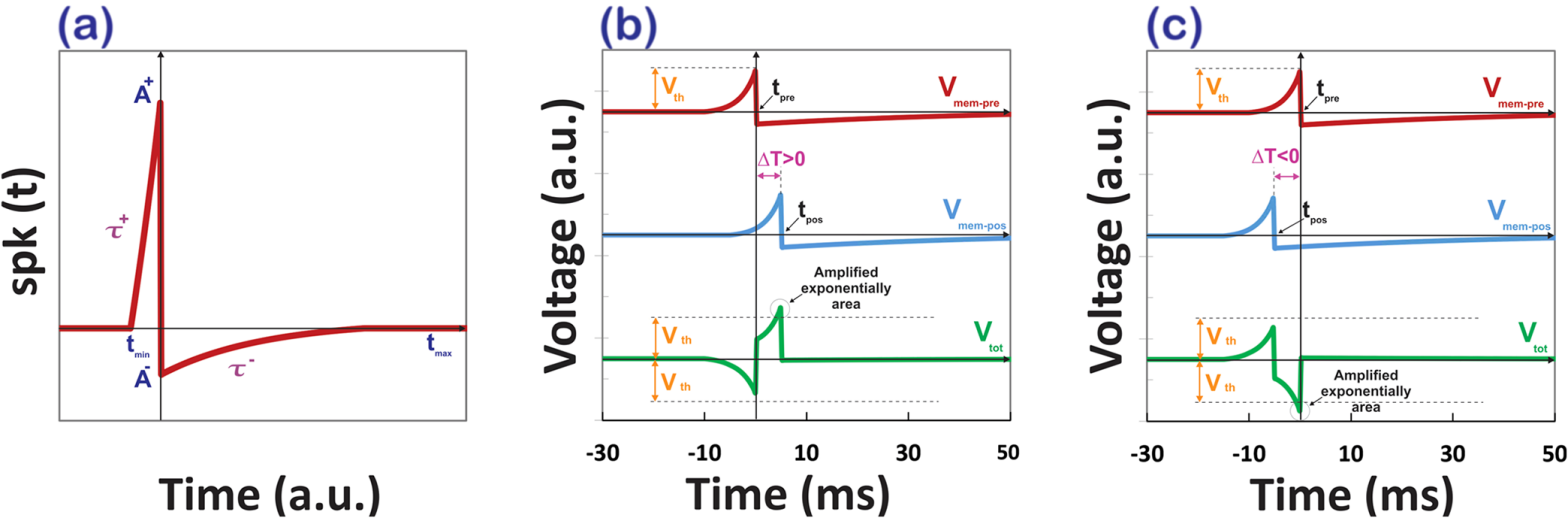
(a) Details of the membrane voltage action potential, as described by eq 7. (b, c) Membrane and memristor dynamics. Pre- and postsynaptic membrane voltages are shown for scenarios with positive Δ*T* (a) and negative Δ*T* (b). The voltage across the memristor, *V*_tot_, is determined by the difference between the postsynaptic membrane voltage (*V*_mem‑pos_) and the presynaptic membrane voltage (*V*_mem‑pre_). When the polarities of these voltages are opposite, their magnitudes combine, potentially surpassing the memristor’s threshold voltage (*V*_th_) defined by the function obtained from eq 3. If this threshold is exceeded, the specified exponentially amplified region drives a change in synaptic efficacy. A positive Δ*T* enhances synaptic efficacy, whereas a negative Δ*T* reduces it.

To STDP with MHP, one must consider the shape of the neural action potentials, or spikes. These action potentials are often difficult to measure precisely, as experimental setups can strongly influence the results. Additionally, different types of neurons produce slightly varying action potential shapes, though they generally share similar characteristics. For the purpose of this discussion, we assume a generic action potential shape with specific properties (as shown in Figure 3a). During the spike onset *t*_min_, the membrane voltage increases exponentially until it reaches a positive peak amplitude *A* ^+^. Following this, the voltage rapidly changes to a negative peak amplitude *A* ^–^ and then smoothly returns to its resting potential during the time *t*_max_.

A mathematical expression can be derived to describe this action potential shape, for instance, by using functions that model the exponential rise and fall in voltage, capturing the distinct phases of the spike. By relating these time-dependent changes in membrane voltage to the behavior of an MHP memristor (whose resistance changes depending on the history of voltage and current), one can connect the dynamics of STDP with the plasticity of memristive devices, where the timing of pre- and postsynaptic spikes modulates the resistance, mimicking synaptic strength changes observed in biological neurons. A shape of the type shown in Figure 3a can be expressed mathematically, for example, as a piecewise function that models the exponential increase and decrease of the membrane voltage during the spike. One possible representation could be

7 $$s\mathrm{pk}\left(t\right)=\{\begin{array}{ll} A^{+}\frac{e^{t/\tau^{+}}-e^{t_{\min}/\tau^{+}}}{1-e^{t_{\min}/\tau^{+}}}, & \mathrm{if}\;t_{\min}<t<0\\ A^{-}\frac{e^{-t/\tau^{-}}-e^{{-t}_{\max}/\tau^{-}}}{1-e^{{-t}_{\max}/\tau^{-}}}, & \mathrm{if}\;0<t<t_{\max}\\ 0 & \mathrm{otherwise} \end{array}$$

where parameters τ^+^ and τ^–^control the curvature of the onset and offset sides of the action potential. Considering pre- and postsynaptic neurons of the same type, they generate the same action potential shape spk (*t*) from eq 7 when they fire. Axons and dendrites act as transmission lines, so some attenuation is expected when the spikes reach the respective synapses. Let α_pre_ be the attenuation factor for the presynaptic spike, represented by *V*_mem–pre_ (*t*) = α_pre_spk (*t* – *t*_pre_), and α_pos_ for the postsynaptic spike, represented by *V*_mem–pos_(*t*) = α_pos_spk (*t* – *t*_pos_). When both spikes are nearly simultaneous at the two cell membranes of the synapse, the ion channels on both membranes open. This suggests a path for substances to move directly between the two cells, which can be modeled using a memristive law similar to those described in eq 3. Thus, we have a two-terminal memristive device between the inside sides of the two cells, specifically between *V*_pos–_ and *V*_pre–_ as shown in Figure S6. The memristor voltage is then *V*_tot_ = *V*_pre–_ – *V*_pos–_. Since the outside nodes of both membranes, *V*_pos+_ and *V*_pre+_, are very close together, both voltages will be approximately equal, as below

8 $$V_{\mathrm{tot}}\left(t\right)=V_{\mathrm{mem}-\mathrm{pos}}\left(t\right)-V_{\mathrm{mem}-\mathrm{pre}}\left(t\right)=\alpha_{\mathrm{pos}}\mathrm{spk}\left(t-t_{\mathrm{pos}}\right)-\alpha_{\mathrm{pre}}\mathrm{spk}\left(t-t_{\mathrm{pre}}\right)$$

By considering Δ*T* = *t*_pos_ – *t*_pre_ eq 8 can be rewritten as

9 $$V_{\mathrm{tot}}\left(t\right)=\alpha_{\mathrm{pos}}\mathrm{sp}k\left(t\right)-\alpha_{\mathrm{pre}}\mathrm{spk}\left(t+\Delta T\right)$$

It is important to note that the chosen voltage pulse shape is not arbitrary, but rather designed to reproduce the characteristic temporal and amplitude dynamics of biological action potentials. The exponential rise and fall phases mimic the depolarization and repolarization of the neuronal membrane, while the threshold alignment (A^+^ ≈ *V*_th_) ensures that conductance modulation occurs only under biologically analogous excitation levels. The correspondence between ionic motion in memristive materials and ion-channel conductance modulation in biological synapses provides the physical basis for this analogy, justifying the use of spike-shaped voltage pulses as a faithful electrical counterpart to neuronal spikes.

Figure 3 shows the membrane and memristor waveforms according to eqs 7–9. The memristor voltage is illustrated in Figure 3b,c for scenarios where Δ*T* is either positive or negative. As outlined in eqs 3–6, the MHP memristor exhibits a threshold voltage (*V*_th_); once this threshold is exceeded, the current through the device experiences a sudden surge. This critical voltage can be defined as the threshold voltage, with the spike amplitude (*A* ^+^) calibrated to match this value. As previously hypothesized, the memristive process facilitates the exchange of a certain quantity of synaptic structural substance(s), denoted as Δ*w*, between the two sides of the synapse. This exchange of Δ*w* ultimately influences the synaptic strength, modulating its efficacy.

Let us define the amount of synaptic structural substance, Δ*w*, exchanged between the two synaptic terminals as governed by the MHP memristive relationships outlined in eqs 1–6. Based on this, the changes in synaptic weight can be expressed as follows

10 $$\Delta w\left(\Delta T\right)=\int I(V_{\mathrm{tot}}\left(t,\Delta T\right))dt$$

The highlighted regions in Figure 3b,c represent the portions of the total memristor voltage, *V*_tot_, that exceed the threshold Δ*T* (for Δ*T* > 0) or fall below −*V*_th_ (for Δ*T* < 0). Within these regions, the memristor current undergoes exponential amplification, as described by the functions in eqs 1–6. When *V*_tot_, exceeds *V*_th_ (for Δ*T* > 0), the corresponding positive areas contribute to an increase in synaptic weight (Δ*w* > 0). Conversely, when *V*_*tot*_ falls below −*V*_th_ (for Δ*T* < 0), the negative areas lead to a decrease in synaptic weight (Δ*w* < 0). As the absolute value of Δ*T*(|Δ*T*|) approaches zero, the peak of *V*_tot_ within these regions becomes more pronounced. Due to the exponential amplification of this peak, the impact on synaptic weight changes—whether incrementing or decrementing *w* becomes significantly stronger as | Δ*T*| decreases.

To investigate the behavior of the MHP memristor, a pulse signal defined by eq 4 was applied to the memristor model in CAD software. The parameters for the pulse were set as Table 2. The resulting output current and current vs voltage were recorded and is displayed in the accompanying Figure 4. The extracted model parameters have clear physical meanings corresponding to halide ion transport and conductive filament evolution. Their values were obtained through dynamic parameter identification across multiple sweep rates, ensuring consistency and physical realism. The excellent agreement between simulation and experiment validates the model’s predictive capability under various electrical stimuli.

**Table 2:** Definition, Unit, and Value of Parameters Describing spk­(t) Signal

parameter	definition	unit	value
*A* ^+^	amplitude for positive voltage	(V)	1.00
*A* ^–^	amplitude for negative voltage	(V)	0.20
τ^+^	time constant for positive voltage	(ms)	12
τ^–^	time constant for negative voltage	(s)	0.05
*t*_min_	exponential spike function start time	(s)	0.05
*t*_max_	exponential spike function end time	(s)	0.3

**Figure 4:**
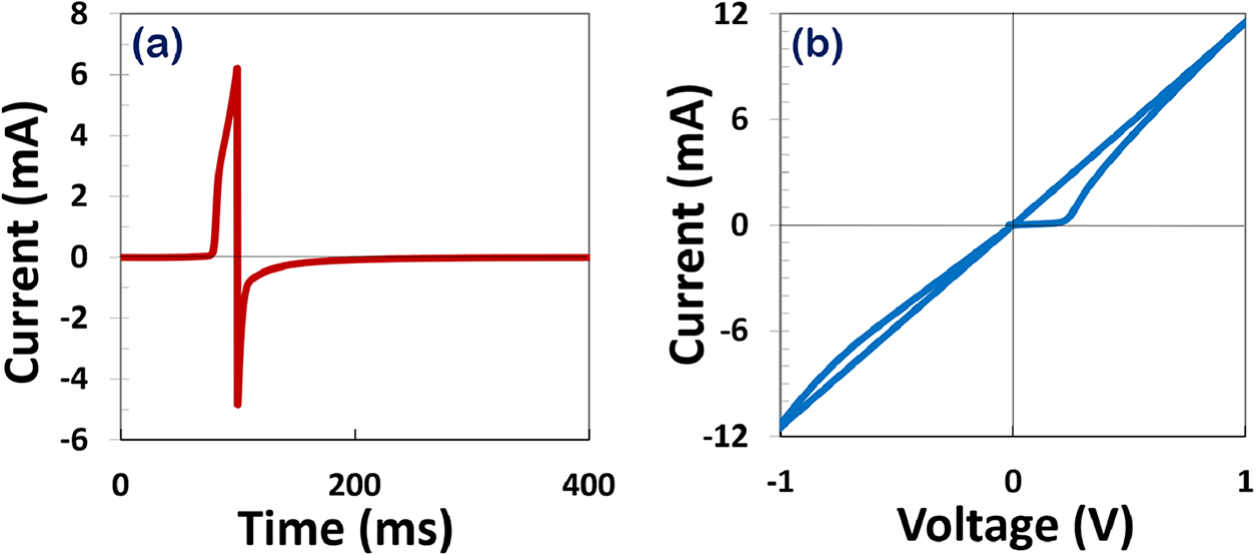
Response of the MHP memristor to a pulsed input signal (defined by eq 4 with parameters from Table 2). (a) The recorded output current over time. (b) The current–voltage (*I–V*) characteristics exhibiting hysteresis, confirming memristive behavior under the applied voltage spike.

As shown in Figure 4b, the memristor’s hysteresis behavior in the *I*–*V* plot is induced by voltages spike inputs. The bipolar resistive switching observed in the Cs_3_Bi_2_I_6_Br_3_ memristor originates from the migration of mobile ionic species within the MHP lattice, primarily halide ions or electrochemically active metal ions, which dynamically modulate the formation and rupture of conductive pathways in the active layer. Under an external electric field, these ions redistribute between electrodes, resulting in reversible transitions between HRS and LRS states. This ion migration process governs the gradual and analog-like modulation of device conductance under pulsed stimuli, enabling continuous tuning of synaptic weight essential for mimicking STDP. The polarity of the applied voltage pulses independently regulates LTP and LTD: a positive bias induces the SET (LTP) process, while a negative bias triggers the RESET (LTD) process. Such bidirectional, polarity-dependent switching behavior mirrors the asymmetric timing dependence in biological synapses, where the order and timing of neuronal spikes determine whether a connection is strengthened or weakened. The gradual conductance evolution under alternating pulses is consistent with partial ion motion, supporting biologically plausible analog plasticity. By exploiting this intrinsic ionic migration mechanism, bipolar memristors can emulate both potentiation and depression within a single device element, enabling efficient and stable STDP implementation without external logic circuits or additional programming overhead. This mechanistic understanding aligns with recent reports that spatially confined ion motion in MHP materials enables low-energy, continuous weight modulation suitable for neuromorphic learning.^41^

The function Δ*w*(Δ*T*), which quantifies the change in synaptic weight, was derived using the memristor model as defined by eq 10. This function, calculated using the memristor model in eq 7, closely aligns with the STDP behavior observed in physiological experiments from the literature. To simulate this, a voltage source based on eq 9 was applied to the MHP memristor. For the numerical computations, the following parameters were utilized: α_pos_ = 1 and α_pre_ = 0.9. By setting α_pos_ ≠ α_pre_, the symmetry of the function ∫*I*(*V*_tot_(*t*,Δ*T*)) is disrupted. Furthermore, when these parameters are significantly distinct, one of the branches of ξ­(Δ*T*) is effectively eliminated. Figure 5a illustrates the STDP behavior derived from a memristive device model. In this analysis, the total voltage applied across the memristive element, *V*_tot_, is defined. The corresponding synaptic weight change Δ*w*(Δ*T*), is extracted by integrating the resulting current through the device over time, yielding a function *f*_Δ*w*_(%) plotted as a function of time interval changes (Δ*T*). This plot reveals the classic STDP profile, characterized by potentiation (positive weight change) for positive Δ*T*, and depression (negative weight change) for negative Δ*T*. The asymmetric shape and exponential decay on both sides of the timing axis are consistent with experimentally observed STDP curves in biological synapses, such as those reported by Bi and Poo.^40^ This result demonstrates that the chosen memristor model, under appropriate spike-based stimulation, inherently supports biologically plausible synaptic plasticity.

**Figure 5:**
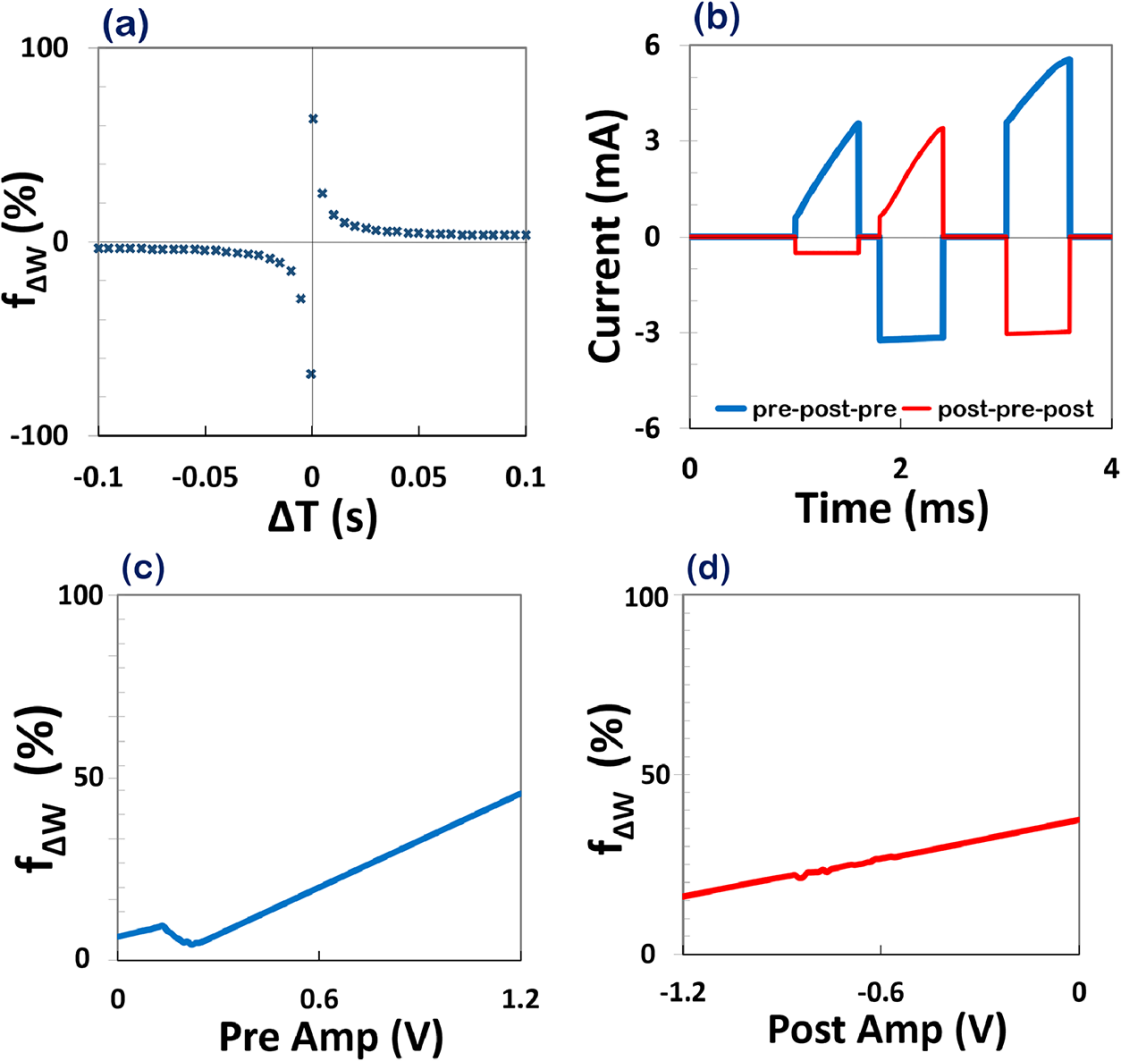
(a) Simulated spike-timing dependent plasticity (STDP) curve derived from a memristive synapse model. The vertical axis represents the normalized change in synaptic weight *f*_Δ*w*_(%), computed as the time integral of the memristor current, while the horizontal axis shows the spike timing difference Δ*T* = *t*_pos_ – *t*_pre_. Positive Δ*T* induces long-term potentiation (LTP), and negative Δ*T* induces long-term depression (LTD), reproducing the typical STDP asymmetry observed in biological systems. (b) Triplet-STDP simulation using a memristor model. The voltage pulse sequences (prepost-pre and postpre-post) induce synaptic potentiation and depression, respectively, demonstrating the dependency of synaptic weight changes on multispike patterns. (c, d) Synaptic weight modulation in a triplet-STDP protocol (pre–post–pre) as a function of spike amplitude.

While classical STDP models describe synaptic changes based on pre- and postsynaptic spike pairs, experimental studies have shown that synaptic plasticity also depends on multispike patterns. Triplet-STDP captures this higher-order dependency by considering sequences such as pre–post–pre and post–pre–post.^42,43^ To understand this behavior of our model, we applied these triplet protocols to a perovskite-based memristor model using tailored voltage pulses. Each spike is implemented same before but in rectangular shape pulse. The use of rectangular voltage pulses in triplet-STDP experiments ensured precise spike timing control, which was essential for isolating the memristor’s intrinsic synaptic behavior. As shown in Figure 5b he pulses are spaced by Δ*T*_1_ = 0.8 ms and Δ*T*_1_ = 1.2 ms, allowing us to model realistic neural triplet timings. Results reveal that in the pre–post–pre configuration, the net current through the device used here as a proxy for synaptic weight increases significantly after the third spike, showing cumulative potentiation. In contrast, the post–pre–post sequence results in an initial small current increase followed by a net decrease, indicating synaptic depression. These behaviors align with biological observations where synaptic modifications depend on both spike order and timing. Such triplet-based dynamics demonstrate that our memristor not only replicates basic STDP, but also supports complex temporal learning rules critical for neuromorphic learning system.

Figure 5c illustrates the relationship between the integrated synaptic current representing the effective synaptic weight and the amplitude of the presynaptic voltage pulse, varied from 0 to 1.2 V while keeping the postsynaptic amplitude constant. The observed trend demonstrates a two-phase increase: a gradual rise in synaptic efficacy for voltages below approximately 0.5 V, followed by a steeper enhancement for higher amplitudes. This nonlinear behavior aligns with the voltage-dependent kinetics of the memristive state variable, as governed by the sigmoidal activation function *x*_ss_(*u*). For lower voltages, the memristor remains in a quasi-linear regime with limited state change, while above the threshold-like region (∼0.5 V), the transition into a high-conductance state accelerates, leading to more pronounced current integration. Figure 5d presents the complementary scenario, where the amplitude of the postsynaptic pulse is swept from −1.2 to 0 V, with the presynaptic amplitude held constant. Here, a monotonic increase in synaptic weight is also observed, which can be attributed to the enhanced contribution of the negative pulse in modulating the memristor’s internal dynamics. Notably, more negative postsynaptic voltages significantly shift the state variable by lowering the potential barrier for charge redistribution, as reflected in the exponential voltage terms in the time constant. Together, these results underline the asymmetric and nonlinear sensitivity of the memristive synapse to spike amplitude, a feature that is not only consistent with experimental observations in oxide- and perovskite-based devices but also critical for implementing biologically plausible learning rules such as triplet-STDP.

In biological synapses, repeated stimulation by spike pairs with a fixed temporal correlation (Δ*T*) leads to the gradual stabilization of synaptic efficacy, a phenomenon known as synaptic consolidation.^40,44^ To examine whether our MHP-based memristor can emulate this behavior, we applied repeated pairs of pre- and postsynaptic voltage pulses (Δ*T* = 1 ms) to the device model and monitored the evolution of the maximum output current, taken as a proxy for synaptic weight. As shown in Figure 6a, the peak current increases nonlinearly with the number of spike pair repetitions (*n*), exhibiting a clear sublinear growth followed by saturation beyond ∼10 trials. This behavior closely mirrors the saturation of long-term potentiation (LTP) observed in biological synapses, where synaptic weights asymptotically approach a maximum due to biochemical constraints and homeostatic regulation.^45^ The observed saturation dynamics suggest that the internal state variable of the memristor, which governs conductance modulation, integrates repeated input activity and stabilizes over time, effectively encoding a form of memory consolidation. This emergent property is not explicitly programmed into the model but arises from the intrinsic dynamics of the voltage-dependent state evolution equation, supporting the hypothesis that memristors can serve as physically grounded analogs of synaptic learning and stabilization. Such characteristics are crucial for neuromorphic hardware, where robust, energy-efficient long-term memory is required in response to repeated sensory or cognitive inputs.

**Figure 6:**
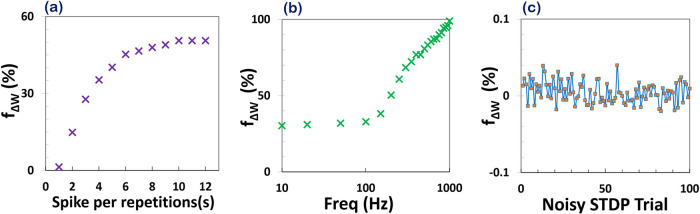
(a) Synaptic weight as peak current vs number of repeated spike pairings (n_trials) with Δ*T* = 1 ms. The memristor exhibits nonlinear current potentiation that saturates after ∼10 trials, indicating synaptic consolidation behavior. (b) Effect of spike-pair repetition frequency on synaptic weight changes in the perovskite memristor. (c) Robustness analysis of synaptic weight change under 100 noisy STDP trials, showing <0.03% variation.

To further investigate the learning dynamics of the memristive synapse, we explored the frequency dependence of synaptic plasticity by applying repeated pairs of pre- and postsynaptic spikes. A total of ten spike pairs were applied at varying repetition frequencies ranging from 10 Hz to 1 kHz. For each frequency, the resultant synaptic weight change was quantified by measuring the final output current of the device, as shown in Figure 6b. The results demonstrate a clear frequency-dependent potentiation effect: as the repetition frequency increases, the final current exhibits a monotonic increase, with a pronounced enhancement observed beyond 500 Hz. This behavior is consistent with frequency-dependent STDP observed in biological synapses, where higher firing rates promote stronger potentiation due to temporal summation and facilitation mechanisms. Such behavior indicates that the perovskite-based memristor not only supports conventional pairwise STDP but also captures higher-order temporal dynamics, such as frequency-modulated plasticity.^22,46,47^ The device demonstrates a key brain-like learning rule (Hebbian learning), highlighting the potential of halide perovskite memristors for creating more biologically realistic neuromorphic computing systems.

To evaluate the robustness and reliability of MHP memristors as artificial synapses, we conducted a noise-resilience analysis by modeling STDP under the presence of biologically inspired voltage noise. In real neural systems, spikes are rarely ideal; they are influenced by biological variability such as thermal fluctuations, timing jitter, and voltage perturbations.^48^ Thus, a truly biorealistic memristive synapse must demonstrate stable STDP characteristics even in the presence of such noise sources. In our simulations, we applied a pair of biphasic prepost voltage pulses to the memristor device, using a fixed Δ*T* and amplitude. From a biological perspective, neuronal spike signals are inherently noisy due to ion-channel stochasticity, background synaptic activity, and thermal fluctuations. Experimental studies report membrane potential fluctuations ranging from a few millivolts up to several tens of millivolts, particularly in vivo where ongoing synaptic bombardment introduces significant variability. Action potential amplitudes, while stereotyped, can also exhibit trial-to-trial variations on the order of 5–20 mV depending on neuron type and firing conditions. To mimic biological noise, we added a normally distributed voltage offset (zero mean, σ = 10 mV) to each time step of the input spike waveform, and repeated the STDP experiment 100 times with independently sampled noise profiles. This noise level was chosen as a conservative estimate of intrinsic neuronal voltage variability and serves as the reference case for the quantitative robustness analysis presented here. Figure 6c summarizes the results of these 100 noisy trials. The vertical axis represents the synaptic weight change (derived from the peak output current) as a percentage relative to the noise-free baseline. The horizontal axis enumerates the trials. As the plot shows, the variation in synaptic weight due to noise remains remarkably small, less than ± 0.03% across all trials, indicating excellent immunity to biologically plausible voltage perturbations. Importantly, the qualitative shape of the STDP curve (LTP/LTD symmetry and polarity) remains intact across all repetitions, with no observable distortion or reversal. These findings support the stability and suitability of the MHP memristor platform for neuromorphic computing applications under realistic operating conditions.

To contextualize the proposed MHP-based synaptic device within the broader literature, Table 3 provides a quantitative comparison with representative perovskite-based memristive synapses previously reported for neuromorphic learning. While earlier studies have successfully demonstrated pair-based STDP in perovskite systems, most reports focus on qualitative learning behavior and do not address higher-order plasticity rules, learning stability under noise, or unified physical modeling. In contrast, the present work demonstrates not only classical STDP, but also triplet-STDP, synaptic consolidation, and exceptional robustness to biologically realistic voltage noise, all captured within a physics-informed compact model suitable for circuit-level simulation.

**Table 3:** Comparison of Perovskite-Based Memristive Synapses Implementing STDP

work	perovskite material	STDP type	noise analysis	weight stability	modeling	key limitation
ref^25^	Cs_3_Cu_2_I_5_	only LTP/LTD under identical pulse trains	not reported	moderate	no	Ag–iodide reaction
ref^17^	hybrid MHP	symmetric and asymmetric STDP	not reported	highly device-dependent	conceptual and system-level modeling	limited endurance and retention
ref^49^	Cu-based (e.g., CsCu_2_I_3_)	asymmetric	not reported	LTP duration up to 40 s; stable over 160 days	not detailed	limited detailed mechanistic study
ref^22^	1D MHP	asymmetric Hebbian STDP	not reported	good long-term retention	DFT modeling and SNN simulation	sneak path issue
ref^50^	CsPbBr_3_	asymmetric	not reported	good reproducibility over many cycles	coupled capacitive-inductive model	needs interface layer
this work	Cs_3_Bi_2_I_6_Br_3_ (lead-free)	pair + triplet-STDP	yes (σ = 10 mV)	<0.03% over 100 trials	physics-based + Verilog-A	—

The insights gained from our STDP simulations suggest that memristive mechanisms, particularly those emerging from halide perovskite materials, can provide a compelling hardware substrate for biologically inspired learning. Beyond mimicking the timing-dependent plasticity observed in neural tissue, these devices demonstrate compatibility with the architectural principles of neuromorphic computing. As illustrated in Figure S7, a hybrid neuromorphic platform can be envisioned in which CMOS-based spiking neurons interface directly with ultradense memristive crossbars functioning as plastic synaptic layers. In this architecture, each neuron drives voltage pulses into the memristor array, inducing conductance changes that encode synaptic weights according to STDP rules. Peripheral CMOS circuitry handles spike generation, read/write operations, and current summation along crossbar columns, allowing asynchronous, event-driven computation. The memristive layer supports local weight updates without global control signals, thereby reducing power consumption while enabling scalable synaptic connectivity. These design considerations demonstrate a practical route for implementing brain-like learning rules in hardware and strengthen the case for memristor-based, low-power neuromorphic computing systems.

## 5 Conclusion

In this work, we have explored the potential of halide perovskite (Cs_3_Bi_2_I_6_Br_3_) memristors as biorealistic synaptic devices capable of implementing spike-timing-dependent plasticity (STDP). Through a combination of experimental measurements, physics-informed modeling, and circuit-level simulation, we established that the MHP memristor exhibits reliable bipolar resistive switching and nonlinear, history-dependent conductance modulation, suitable for mimicking essential synaptic behaviors. Using experimentally derived parameters, we developed a time-continuous model that accurately captures the dynamics of the memristor under paired voltage pulses. This model allowed us to simulate classical pairwise STDP, demonstrating the expected asymmetric learning window, with potentiation and depression governed by spike timing (Δ*T*). We extended this analysis to triplet-STDP protocols, where the device successfully emulated higher-order plasticity rules observed in biological systems. Moreover, we examined memory consolidation by applying repeated spike-pair stimulation, showing that the synaptic weight evolution follows a saturating nonlinear trajectory, closely aligning with long-term potentiation dynamics in biological synapses.

Crucially, we assessed the robustness of the STDP response under realistic noise conditions. By performing 100 independent STDP trials with normally distributed voltage noise (σ = 10 mV, shown in Figure 6c), we observed that the synaptic weight variation remained below 0.03%, confirming excellent noise tolerance. This suggests that the intrinsic nonlinearity and thresholding behavior of the MHP memristor effectively filters out biological-scale perturbations, supporting its viability in noisy neuromorphic environments. Taken together, these results demonstrate that halide perovskite memristors provide a compact, stable, and noise-resilient platform for implementing time-dependent synaptic learning. The compatibility of MHP materials with low-temperature and solution-based processing further enhances their appeal for scalable neuromorphic integration. Future directions include hardware implementation of memristor-based STDP arrays, exploration of temporal learning in spiking neural networks (SNNs), and long-term stability testing under continuous spiking activity.

## Supplementary Material



## Data Availability

The data that supports the findings of this study are available in the supporting data of this article.

## Notes

Fabrication process of Cs_3_Bi_2_I_6_Br_3_MHP memristor (Figure S1); *I–V* characteristics of Au/Cs_3_Bi_2_I_6_Br_3_MHP memristor/ITO device (Figure S2); reproducibility and variability analysis including HRS, LRS, and ON/OFF ratio statistics (Figure S3); device endurance performance (Figure S4); experimental and simulated *I–V* characteristics at different scan rates (Figure S5); schematic illustration of synaptic action and communication (Figure S6); neuromorphic computing architecture integrating STDP-enabled memristors and CMOS neurons (Figure S7) (PDF)
