# Supplementary material for: Bio-Inspired Spike-Timing-Dependent Plasticity Learning with Metal Halide Perovskites: Toward Artificial Synaptic Functionality
Source: ACS Appl Mater Interfaces. 2026 Jan 22;18(4):7103–14. doi: 10.1021/acsami.5c21545 (PMC12884454; doi:10.1021/acsami.5c21545)
Supplement: Supplementary file 1 [file am5c21545_si_001.pdf]

Supplementary Data:

Bio-Inspired Spike-Timing-Dependent Plasticity Learning with Metal Halide Perovskites: Toward Artificial Synaptic Functionality

Mostafa Shooshtari[1*], So-Yeon Kim[2*], Saeideh Pahlavan[1], Teresa Serrano-Gotarredona[1], Juan Bisquert[2*], Bernabé Linares-Barranco[1]

[1] *Instituto de Microelectrónica de Sevilla, IMSE-CNM, (CSIC Universidad de Sevilla), 41092, Sevilla, Spain.*

[2] *Instituto de Tecnología Química (ITQ). Universitat Politècnica de València- Consejo Superior de Investigaciones Científicas (UPV-CSIC). 46022 València, Spain.*

**Corresponding author: J. Bisquert (jbisquer@itq.upv.es), M.Shooshtari (m.shooshtari@imse-cnm.cisc.es) and S.-Y. Kim (skim3@itq.upv.es)*

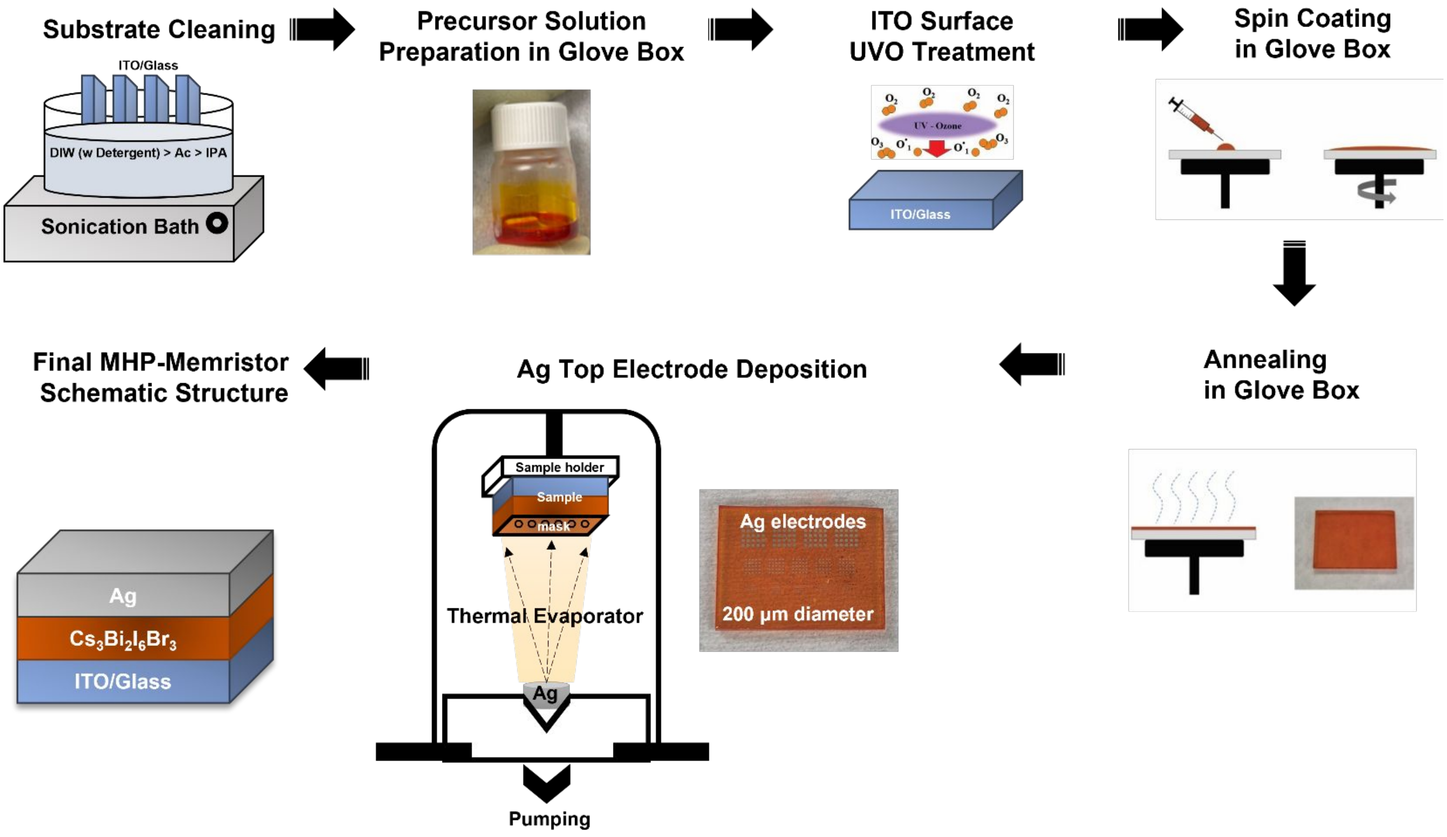

Figure S1: Fabrication prosses of $Cs_3Bi_2I_6Br_3$ MHP-memristor.

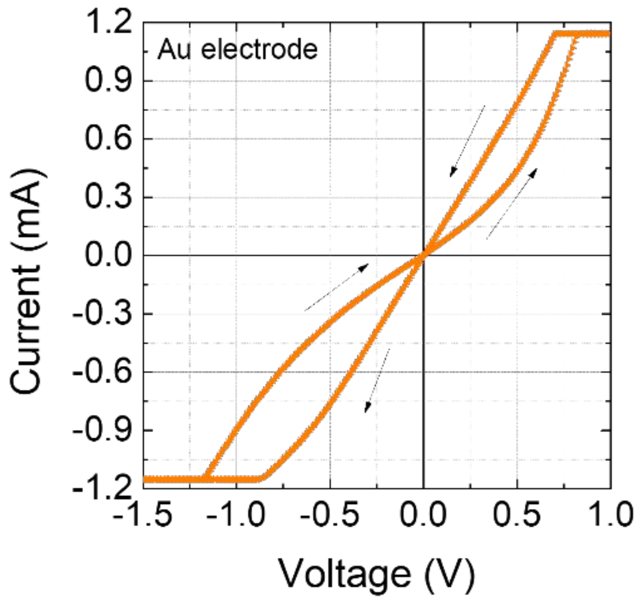

Figure S2: I-V characteristics of Au/$Cs_3Bi_2I_6Br_3$ MHP-memristor/ITO device.

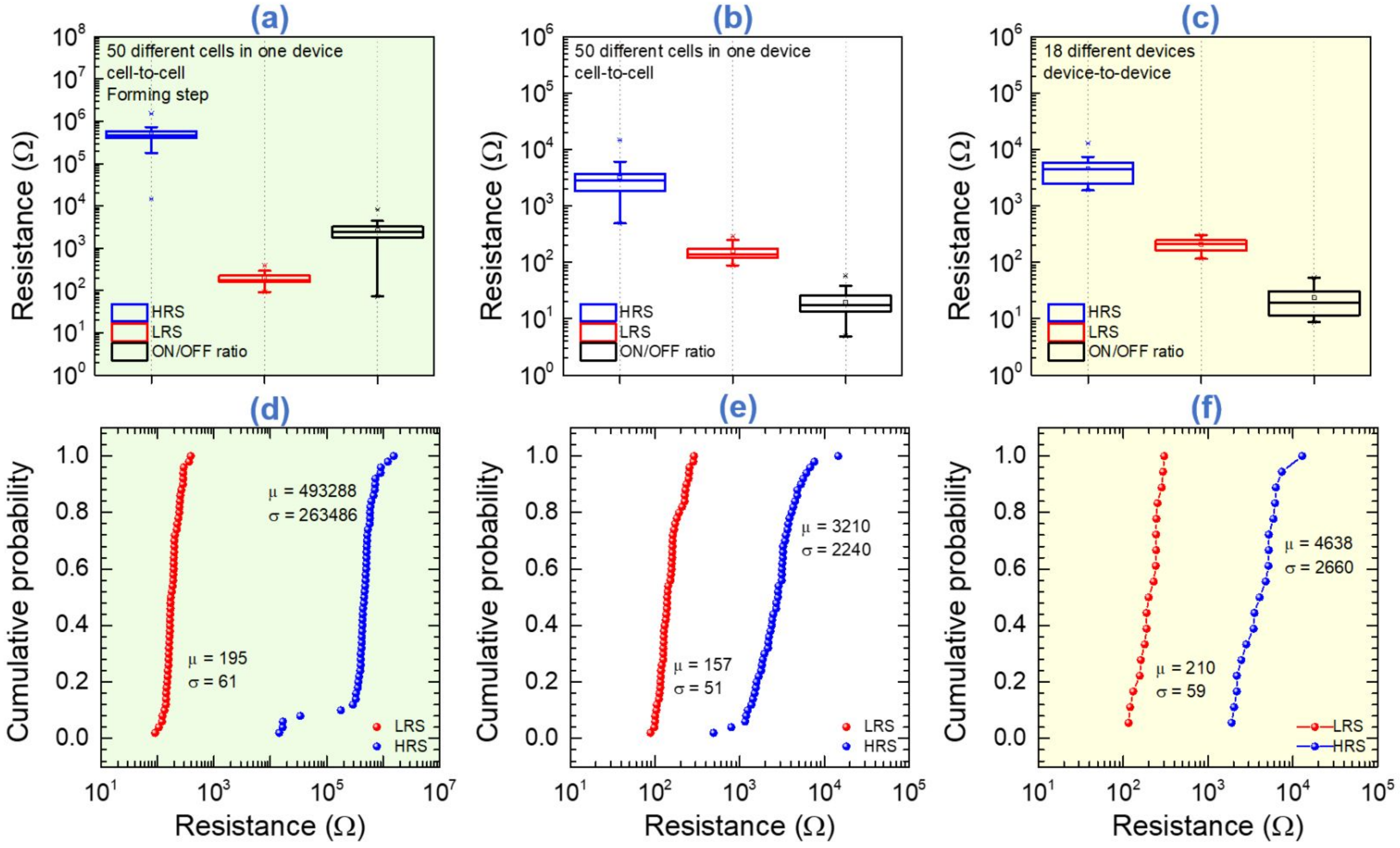

Figure S3: Reproducibility and variability analysis of MHP memristor devices. (a-c) Static box plots of the HRS, LRS, and ON/OFF ratio collected from: (a-b) 50 different cells across a single device (cell-to-cell reproducibility), and (c) 18 cells from different devices (device-to-device reproducibility). (d-f) Corresponding cumulative probability distributions for the (d) HRS, (e) LRS, and (f) ON/OFF ratio, demonstrating low variability. The mean (μ) and standard deviation (σ) values are provided for each distribution.

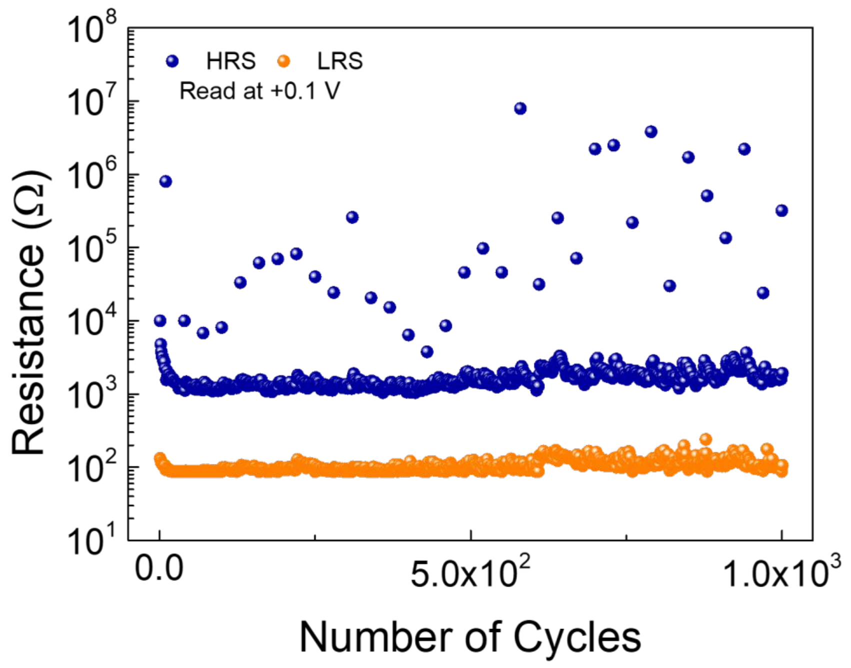

Figure S4: Device durability test (endurance test). The MHP device was applied with +1.0 V for SET processes and -1.5 V for RESET processes and HRS and LRS were read at +0.1 V during 10^3 consecutive repeating cycling tests.

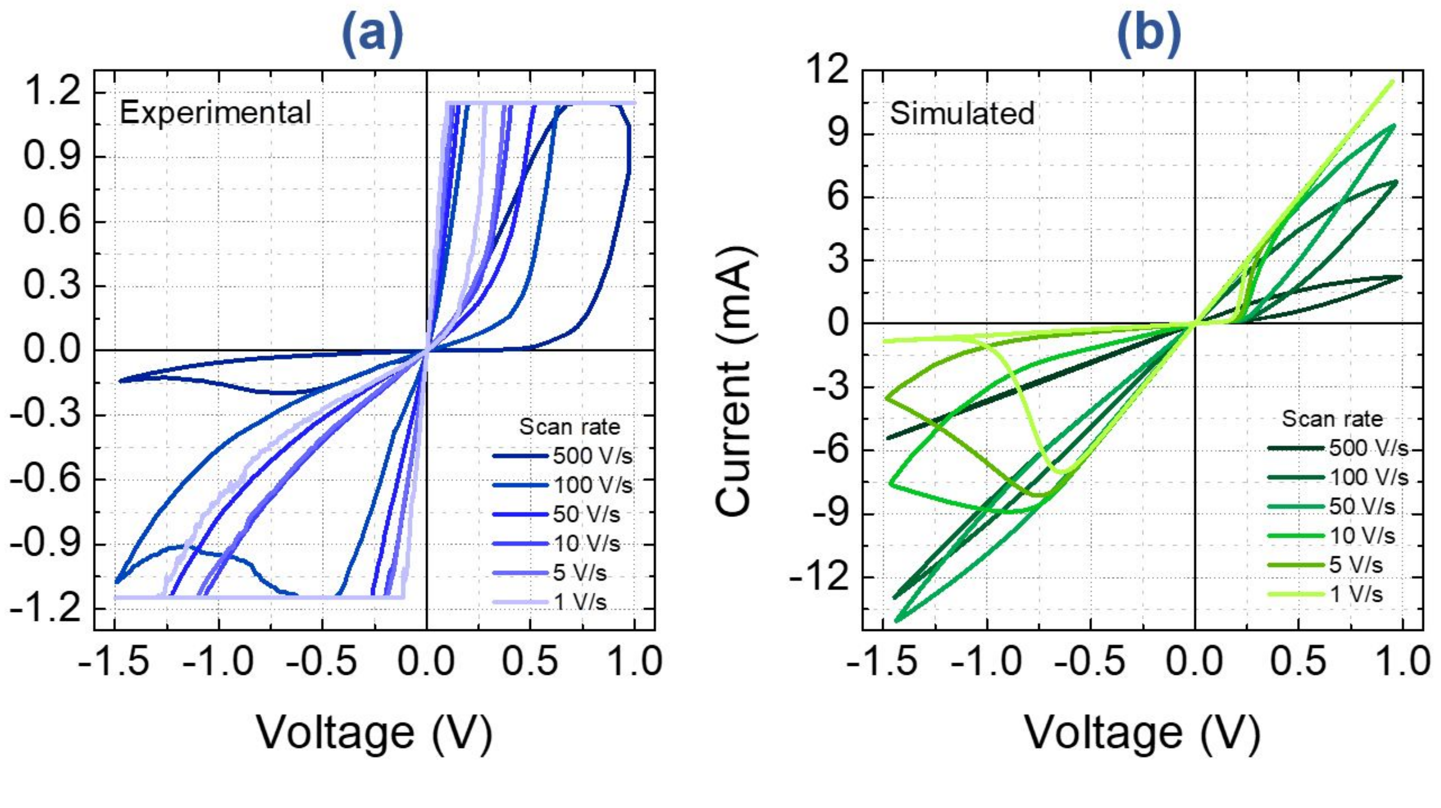

Figure S5: (a) Experimental and (b) Simulated I-V characteristics of Ag/$Cs_3Bi_2I_6Br_3$ MHP-memristor/ITO device depending on the scan rates.

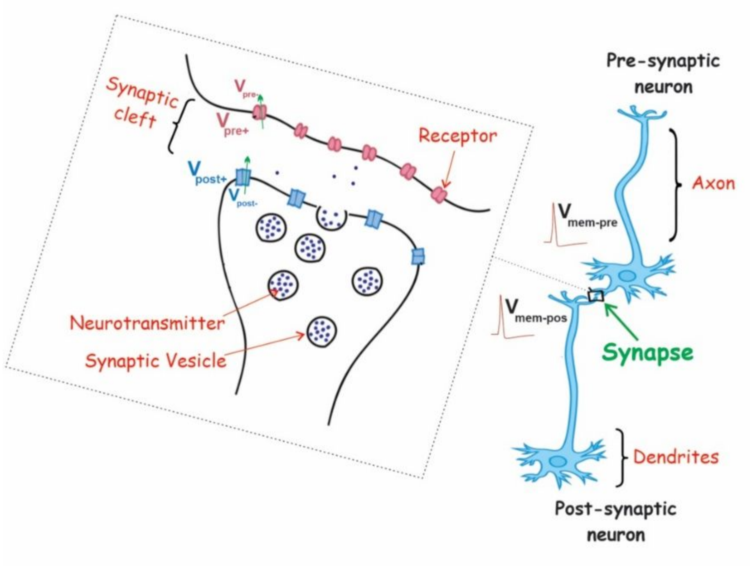

Figure S6: Synaptic Action and Communication: Illustration of synaptic activity, where a pre-synaptic neuron connects with a post-synaptic neuron. The pre-synaptic neuron sends an action potential ($V_{mem-pre}$) through its axon to the synapse. The combined effect of multiple pre-synaptic action potentials generates a post-synaptic action potential, which propagates through the post-synaptic neuron's terminals. The Zoom area shows details of the synaptic junction that membrane channels open and close in response to voltage changes, and neurotransmitter-filled vesicles are released into the synaptic cleft during a pre-synaptic action potential.

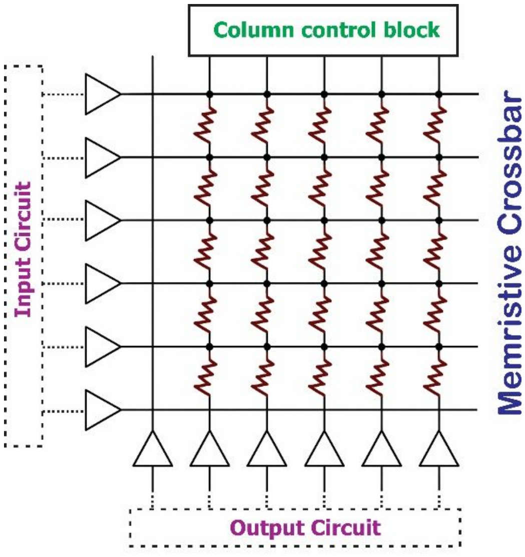

Figure S7: Neuromorphic computing architecture integrating STDP-enabled memristors and CMOS neurons. The system features three spiking neural layers implemented in CMOS technology, interconnected by two fully connected crossbar arrays of memristive devices fabricated with nanowire structures on top of the CMOS substrate. STDP learning is realized by propagating neuronal action potentials forward with full amplitude and backward with controlled attenuation, enabling synaptic plasticity driven by spike timing.
